# The contribution of mechanical ventilation to critical illness-associated diaphragm dysfunction: a critical appraisal

**DOI:** 10.1186/s13054-026-06003-y

**Published:** 2026-04-20

**Authors:** Tommaso Rosà, Jonne Doorduin, Domenico Luca Grieco, Massimo Antonelli, Coen A. C. Ottenheijm, Leo Heunks

**Affiliations:** 1 Department of Anesthesiology and Intensive Care Medicine, Catholic University of the Sacred Heart, Rome, Italy; 2 Department of Emergency, Intensive Care Medicine and Anesthesia, Fondazione Policlinico Universitario A. Gemelli IRCCS, Rome, Italy; 3 Department of Intensive Care Medicine, Radboud University Medical Centre, Geert Grooteplein Zuid, Nijmegen, 6525 HP The Netherlands; 4 Department of Physiology, Amsterdam UMC, Location VUmc, Amsterdam, The Netherlands

## Abstract

Although diaphragm dysfunction is increasingly recognized as a major contributor to morbidity and mortality in the intensive care unit, the physiological mechanisms leading to its development in mechanically ventilated patients are incompletely understood. Mechanical ventilation is often recognized as the main cause of the problem, delineating a paradigm known as *ventilator-induced diaphragm dysfunction* (VIDD). Yet, evidence on this causal relationship in ventilated critically ill patients is scarce, with direct experimental data mostly coming from animal models or brain-dead organ donors. In this narrative review, we provide a critical appraisal on the contribution of mechanical ventilation to diaphragm dysfunction and an integration with other major mechanisms involved in its development and trajectory over time. Examining this complex interplay is important, as it may support clinicians in adhering to expert consensus on diaphragm protective mechanical ventilation. First, the evidence for mechanisms potentially caused by mechanical ventilation, such as disuse atrophy, under- and over-assistance, PEEP and asynchronies is analyzed. Secondly, important contributors not directly explained by ventilator support, such as inflammation and sepsis, muscle hibernation and impaired calcium sensitivity of force are addressed. Finally, a summary of this complex scenario is provided together with clinical and research-oriented key messages, highlighting the reasons for which the term *critical illness-associated* diaphragm dysfunction may be more appropriate.

## Introduction

Every clinician in the intensive care unit (ICU) or scientist will agree that critically ill patients are at risk of developing respiratory muscle weakness, and that this is associated with difficult ventilator weaning [1–6]. However, the physiological mechanisms underlying weakness in mechanically ventilated patients are incompletely understood. Many clinicians and scientists pinpoint the ventilator as the main driver of development of diaphragm weakness [7–9], defining this phenomenon as *ventilator-induced diaphragm dysfunction (VIDD)* [7, 10–13]. This presumed causal relationship between mechanical ventilation and diaphragm dysfunction introduced the concept of *diaphragm protective* mechanical ventilation, which has gained rapid popularity in recent literature [14–19]. The key recommendation of this concept is that inspiratory support should be titrated to allow physiological levels of diaphragm effort (respiratory muscle pressure, Pmus, between 3 and 12 cmH_2_O) and avoid under- and over-assistance. However, before implementing such approach in clinical practice, it is important to understand the role of mechanical ventilation in the development of diaphragm weakness in critically ill patients, and to quantify its attributable and/or possibly modifiable damaging component. This may help prioritize diaphragm protection over potentially conflicting goals such as lung protection and prevention of brain dysfunction (delirium, altered cognition) resulting from increased use of sedatives. The aim of this narrative review is to provide a critical appraisal on the role of mechanical ventilation in the pathophysiology of diaphragm dysfunction in mechanically ventilated critically ill patients. This may allow the identification of future research avenues in the field and, more importantly, guide clinicians upon the role of diaphragm protective ventilation in clinical decision making.

## Mechanical ventilation and diaphragm dysfunction

In critically ill patients, it is difficult to disentangle the contribution of mechanical ventilation in the development of diaphragm weakness, as multiple concurrent factors may confound this causal relationship. Here, we show potential mechanisms that contribute to critical care associated diaphragm dysfunction based on preclinical and human studies.

### Diaphragm disuse atrophy

**Fig 1.**
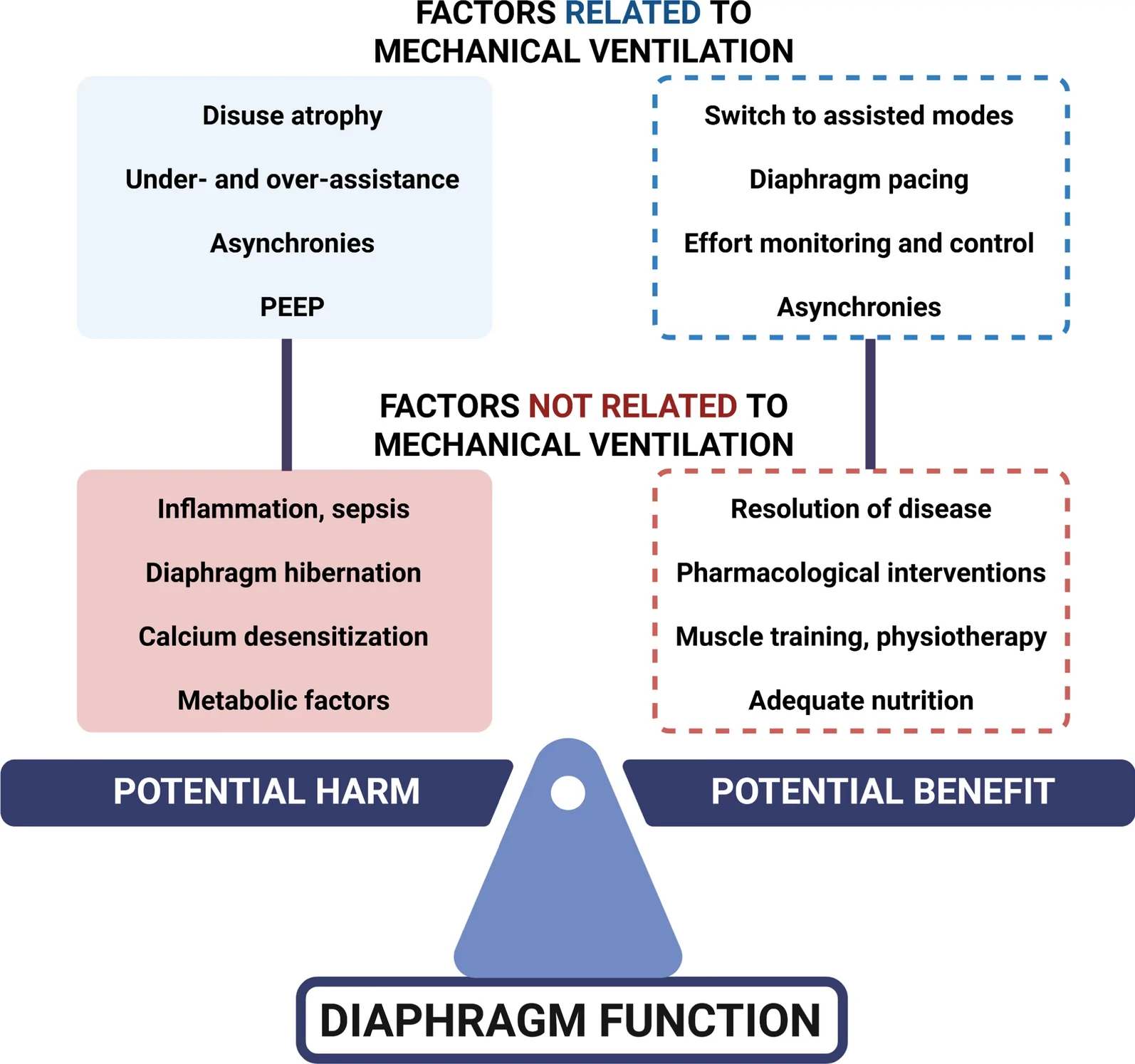
Conceptual balance between mechanical ventilation- and critical illness-related factors involved in the modulation of diaphragm function. On the left side of the figure, mechanisms thought to negatively affect diaphragm contractility and efficiency are listed (colored boxes), whereas those potentially playing a beneficial role are shown on the right side of the conceptual balance (dashed boxes). All mechanisms related with and modulated by mechanical ventilation are shown on the top (blue color), while red boxes highlight the role of modulators unrelated to mechanical ventilation. Of note, asynchronies are included on both sides of the balance, as patient-ventilator interaction is thought to possess a double-edged nature in terms of risk of myotrauma, as in the case of reverse triggering

Consistently with basic muscle physiology, prolonged mechanical unloading of respiratory muscles induces wasting. This paradigm is part of the broader framework of critical-illness myopathy [20], whereby prolonged immobilization, sedation, as well as inflammation and critical illness per se act synergistically promoting muscle wasting. The so-called diaphragm disuse atrophy is a recognized consequence of prolonged mechanical ventilation (Fig. 1).

#### Evidence from animal models

Controlled mechanical ventilation in rats leads to a time-dependent reduction in diaphragm muscle fiber cross-sectional area, with atrophy occurring after 12-18 hours [11, 21, 22]. This phenomenon is mechanistically linked to a depression in protein synthesis [23], particularly of myosin heavy chain, without a corresponding decrease in myosin mRNA, indicating post-transcriptional regulation [24]. Atrophy results in reduction of force-generating capacity, both *in-vitro* and *in-vivo* [25–27]. Furthermore, recovery studies in animal models show that diaphragm reloading after controlled mechanical ventilation can partially restore force and fiber dimensions within hours, but complete recovery requires longer periods [28].

#### Evidence from ventilated patients

The relationship between diaphragm disuse atrophy and mechanical ventilation in humans is complex. Histopathological studies conducted in brain-dead organ donors provided direct evidence of rapid structural degradation of diaphragm myofibers [29–31]. In a landmark study, Levine and colleagues demonstrated marked muscle fiber atrophy consisting of a decrease in cross-sectional area of 57% for slow-twitch fibers, and of 53% for fast-twitch fibers as compared with controls. Brain-dead cases had diaphragmatic inactivity and were ventilated for 18 to 69 hours, and atrophy resulted from activation of proteolytic and autophagic pathways [32]. Other authors confirmed a cross-sectional area reduction of all diaphragm fibers averaging 39% [31]. Conversely, Hooijman and coworkers found no differences in fibers cross-sectional area nor in force generation between brain-dead cases and controls, although the mean duration of mechanical ventilation was slightly shorter than in previously mentioned studies (“only” 26 hours) [33]. Nevertheless, the generalizability of these data is limited, as brain-dead organ donors represent a unique patient category, with potentially limited reproducibility. Moreover, most of the brain dead patients studied had traumatic brain injury, which may directly impact muscle physiology [34]. In a group of mechanically ventilated critically ill patients not including brain-dead organ donors, fiber atrophy was less pronounced (25%-reduction in fiber cross-sectional area), and was insufficient to fully explain the observed severe functional weakness [35]. Importantly, sarcomeric protein dysfunction, related to the upregulation of the ubiquitin-proteasome pathway, was shown to play a crucial role in this patient group, together with reduced calcium sensitivity of force generation [35–38]. Therefore, the presence of sarcomeric protein dysfunction, whereby fibers are less efficient in generating contractile force, helps explain the observed severe weakness despite modest degrees of muscle atrophy. The existence of sarcomere dysfunction and its contribution to muscle weakness was confirmed by the same group proving that individual myofibers force *deficit* may persist after normalization for cross-sectional area [36]. Finally, differently than in organ donors [30], diaphragm biopsies from mechanically ventilated critically ill patients showed substantially preserved mitochondrial structure and function [37].

*In vivo* detection and monitoring of diaphragm atrophy during critical illness is also challenging. Ultrasound has been used to measure diaphragm thickness and thickening fraction at the bedside, and quantify atrophy and contractility [39]. At population-level, both reductions and increases in thickness over time are associated with worse clinical outcome in mechanically ventilated patients [4]. However, ultrasound measurements may be influenced by patient positioning, anatomy, probe selection and ventilator settings, and the correlation with transdiaphragmatic pressure is poor [40]. This leads to variability in thickness values interpretation at patient-level. Furthermore, ultrasound cannot directly measure diaphragm force generation or fatigue [40, 41], and the association between diaphragm thickness and atrophy may be influenced by various confounders, such as extracellular matrix accumulation (i.e. replacement fibrosis) [42].

### Load-induced diaphragm injury

Load-induced diaphragm injury, or myotrauma, refers to diaphragm structural and functional damage caused by increased inspiratory effort or mechanical load that exceeds physiological capacity and yields fiber disruption, inflammation, and impaired contractility [43, 44] (Fig. 1).

#### Evidence from animal models

Animal models provided foundational insights into load-induced diaphragm injury, yet their translation to critically ill patients remains challenging. Early experimental works demonstrated that resistive inspiratory loading can cause structural injury [44, 45]. In these animal models, loading protocols were intense: Zhu et al. targeted peak negative inspiratory tracheal pressure values in the range of -30 to -50 cmH2O for two hours per day over 24 consecutive days in dogs; Reid et al. increased airway resistance in hamsters through tracheal banding for 6 days, with a 66%-reduction in tracheal cross-sectional area [44]. Similarly, Jiang et al. showed that in rabbits, load-induced injury occurred only when inspiratory resistive loads exceeded the fatigue threshold, highlighting that injury is contingent on extreme loading conditions [46]. Finally, a study in rats showed that histological damage associated with resistive loading seemed to peak around day three and partially resolve by day four, suggesting a role of remodeling [47]. These studies, however, relied on intense resistive loads in previously unventilated animals, questioning the reproducibility of findings in patients. More recent evidence in piglets by Capdevila et al. suggests that ventilator under-assistance with greater dyssynchrony rate following prolonged controlled ventilation worsens diaphragm function and favors ultrastructural injury [48]. On the contrary, the same conditions seemed not to affect the diaphragm of animals that were not previously ventilated. Intriguingly, the magnitude of inspiratory effort did not correlate with structural damage, as effort was importantly lower in the animal group receiving prolonged mechanical ventilation (where diaphragm injury was greater). Such findings challenge the causal relationship between inspiratory effort and myotrauma. Furthermore, the brief, two-hour exposure of animals to under-assistance raises questions about the potential contributions of fatigue [49, 50] and muscle remodeling on a longer term.

**Fig 2.**
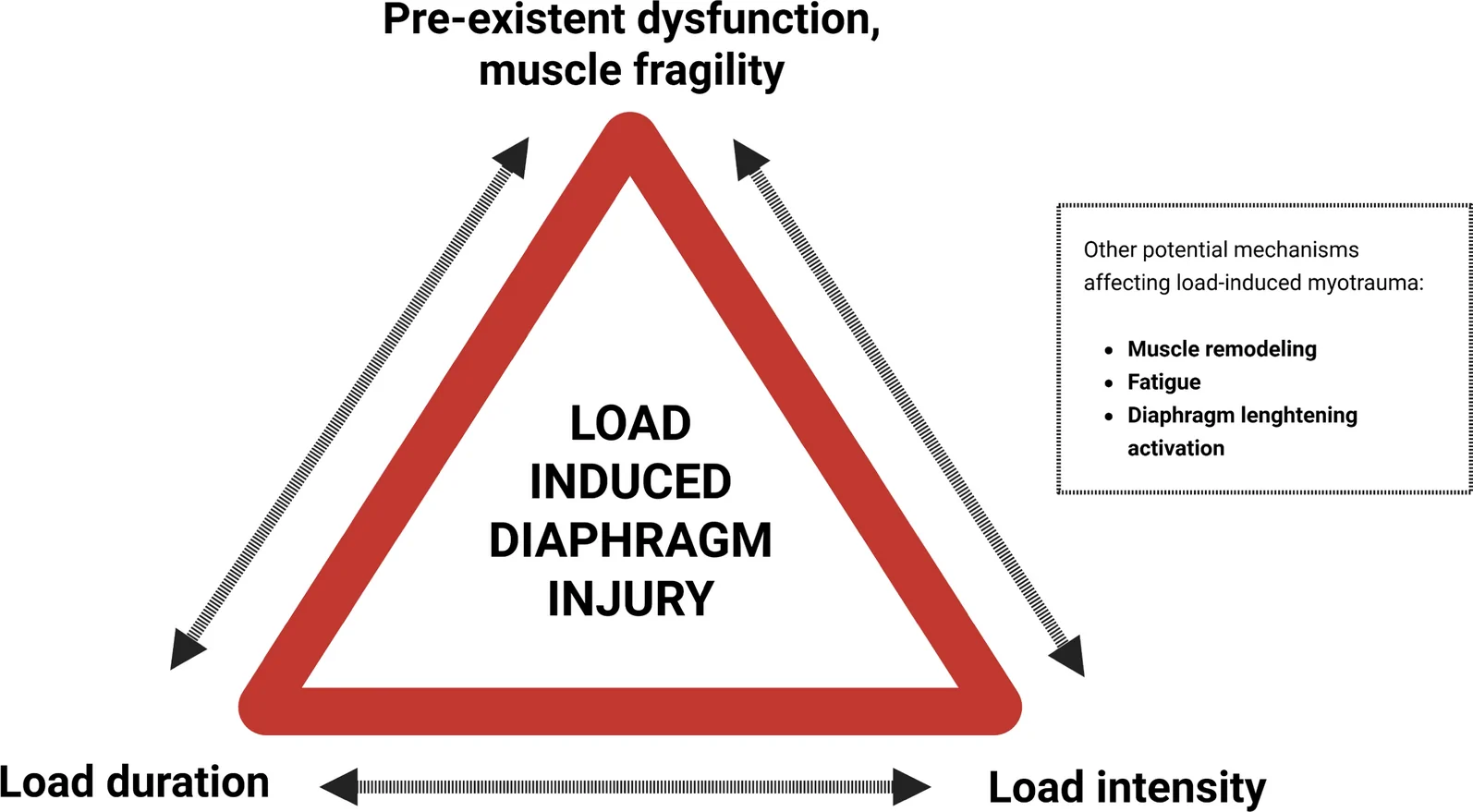
Determinants of load-induced diaphragm injury. The figure depicts the interplay between load intensity, load duration and baseline diaphragm function for the development of load-induced myotrauma. The final impact of each of the three main determinants is directly influenced by the other two, supporting the double (or triple) hit model theory for diaphragm injury development during spontaneous/assisted breathing. Muscle remodeling, fatigue and diaphragm lengthening activation are mentioned as further potential contributors to injury

Collectively, these studies highlight that although excessive inspiratory loading can injure the diaphragm in animals, the impact and mechanisms involved may differ substantially according to multiple variables such as ventilation history, duration, breathing mechanics (e.g. effort magnitude), and subject-ventilator interaction (Fig. 2).

#### Evidence from ventilated patients

The concept of load-induced diaphragm injury during spontaneous breathing and assisted mechanical ventilation, although physiologically sound, remains poorly demonstrated in critically ill patients for obvious technical reasons. Early experimental studies provided the foundation for this hypothesis: Orozco-Levi and colleagues showed that inspiratory loading to 80% (!) of maximal threshold pressure produced ultrastructural modifications consistent with diaphragm injury in healthy subjects and, to a greater extent, in patients with COPD, suggesting that excessive inspiratory efforts can be harmful for the diaphragm [51]. Similarly, Briskey et al. observed an increase in markers of oxidative stress in plasma of healthy subjects only after loading to 70% of subjects’ maximum transdiaphragmatic pressure [52]. Critically ill patients have reduced muscular reserve, impaired diaphragm function and multiple, intense *stimuli* for elevated respiratory drive [2, 53]: hence, they may reach their load-injury threshold relatively early. However, the association between high effort and diaphragm myotrauma has never been demonstrated in this population, and it is not easy to predict the timing nor the threshold for such phenomenon to occur. Importantly, mechanically ventilated critically ill patients are seldom exposed to inspiratory loading of comparable extent to that evaluated in experimental studies.

### Positive end-expiratory pressure

Positive end-expiratory pressure (PEEP) has several, complex effects extending beyond alveolar pressure modifications [54–58], including changes in diaphragm geometry and function [15]. Shortly, these effects can be attributed to changes in lung volume, which in turn affect the position and shape of the diaphragm, ultimately modulating its efficiency (Fig. 1).

#### Evidence from animal models

Morais et al. first evaluated the effects of increasing PEEP on respiratory mechanics during assisted breathing in a mixed population of rabbits and pigs with experimentally induced ARDS [59]. In their study, higher PEEP consistently reduced inspiratory effort: the authors explained such finding hypothesizing that the PEEP-induced caudal displacement of the diaphragm negatively affected its efficiency by means of electromechanical uncoupling. This would occur through a shortening of muscle fibers in the zone of apposition, placing sarcomeres at a less favorable operating length in the force-length relationship. This hypothesis was later confirmed in an experimental study in pigs [60], and aligns with the long known relationship between increased lung volume and reduced diaphragm efficiency [61]. On the other hand, experimental animal work indicates that the diaphragm may adapt to the altered configuration caused by prolonged exposure to PEEP [62]. In particular, fibers in the zone of apposition can shorten by expelling sarcomeres arranged in series, which helps the muscle return to an optimal force/length relationship. This adaptation, where the predominant change is a reduction in muscle length due to loss of sarcomeres in series, is termed *longitudinal atrophy*, as it differs from classic cross-sectional atrophy where the muscle becomes thinner due to loss of sarcomeres in parallel. This mechanism has potential relevance during weaning: indeed, when PEEP is abruptly discontinued for a spontaneous breathing trial, end-expiratory lung volume falls and a longitudinally shortened diaphragm is stretched, worsening actin–myosin alignment and limiting force production. Finally, evidence suggests that prolonged application of PEEP may trigger further structural changes: in rabbits undergoing mechanical ventilation for 48 hours, PEEP was linked to increased collagen deposition and diaphragm fibrosis [63].

#### Evidence from humans

The effects of PEEP on lung and diaphragm physiology have been evaluated in healthy subjects and hypoxemic ICU patients [18, 59, 64–67].

In healthy volunteers, magnetic resonance imaging (MRI) confirmed that the PEEP-induced increase in lung volume shifted the diaphragm caudally and shortened the length of its zone of apposition. This was accompanied by reduced pressure generating capacity and impaired neuromechanical efficiency of the diaphragm [61, 66]. Morais et al. proposed a similar explanation for their findings in patients with ARDS, where high PEEP uniformly decreased effort without changing electrical activity of the diaphragm [59]. Importantly though, patients in this study were either monitored solely with esophageal manometry or diaphragm electromyography, with the two never measured simultaneously. On the other hand, later works highlighted a greater degree of heterogeneity in PEEP-induced changes in inspiratory effort among critically ill patients [18, 64, 67], a population in which diaphragm neuromechanical efficiency at varying PEEP levels has never been formally assessed. This heterogeneity could be explained by variable effects of PEEP on respiratory system compliance, lung overdistention, caudal displacement of the lower ribs, gas exchange and expiratory muscles recruitment [18, 64, 65, 67, 68].

Finally, to date, no data is available demonstrating the occurrence of longitudinal atrophy or any other diaphragm remodeling process induced by PEEP in ventilated patients.

### Patient-ventilator (a)synchrony

Asynchronies and diaphragm lengthening activation (often referred to as eccentric contraction, during which fibers lengthen while activated) have been proposed as contributors to diaphragm myotrauma (Figs. 1 and 2). Lengthening activations, however, often occur during various physiological conditions (e.g. physical exercise) where they promote muscle growth and repair, and therefore are not necessarily harmful to the muscle, particularly when of modest intensity [69].

#### Evidence from animal models

The risks of diaphragm lengthening activation relate to the damage resulting from muscle activity while muscle fibers are lengthening, typically during expiration. Pellegrini and colleagues monitored expiratory electrical activity of the diaphragm in a porcine model of ARDS [40]. In their experiments, lowering PEEP increased expiratory diaphragm activity, consistently with a diaphragm ‘breaking’ effect against the loss of lung volume. Conversely, suppressing this activity with sedation and neuromuscular blockade led to more atelectasis, supporting the idea that expiratory diaphragm recruitment helps limit tidal derecruitment. However, diaphragm lengthening activation has been hypothesized to have mixed effects on diaphragm integrity [70]: compared with concentric contractions, it imposes greater strain on muscle fibers leading to myotrauma, yet it may also promote muscle remodeling. Diaphragm lengthening activation is a phenomenon that occurs during certain asynchronies as well, particularly reverse triggering, with the risks on lung and diaphragm physiology being seemingly related to the magnitude of the generated contraction [71–73]. Finally, Hashimoto and colleagues reported a higher frequency of diaphragm lengthening activations in rabbits ventilated with breath stacking. In the same animals, force-generating capacity was reduced and markers of diaphragmatic injury were more pronounced [73]. Interestingly, evidence by Damiani et al. showed that low levels of inspiratory effort induced by reverse triggering may help preserve diaphragm function in ventilated pigs [72]. This finding suggests a possibility whereby diaphragm activity especially characterized by lengthening activation may counteract disuse atrophy and serve as an effective ‘training’ *stimulus*, similarly to what occurs in other skeletal muscles [74–76].

#### Evidence from ventilated patients

In critically ill patients receiving mechanical ventilation, diaphragm lengthening activation is frequent and often occurs during post-inspiratory activity [77, 78] or patient-ventilator asynchronies such as ineffective efforts, premature cycling, and reverse triggering [70, 79, 80]. Post-inspiratory diaphragm activity occurs during exercise or inspiratory loading in healthy subjects as well [77], and can therefore be considered (at least to a certain extent) a physiological phenomenon. The clinical implications may be significant: optimizing ventilatory support to minimize diaphragm lengthening activation is intriguing, and a bedside evaluation of breathing effort is recommended to guide interventions. However, the impact of the phenomenon, as well as that of asynchronies, on long-term outcomes remains debated. Notably, two recent clinical studies have associated the presence of reverse triggering with a higher likelihood of successful transition to assisted ventilation or extubation and reduced mortality among patients with ARDS, suggesting a potential benefit related to this particular asynchrony [80, 81]. The consequences of asynchronies may therefore depend on their nature, their repercussions on lung and diaphragm physiology and on specific patient categories. For example, wasted efforts may serve as a training stimulus for the diaphragm of mechanically ventilated patients, and reverse triggering may be less dangerous in COPD than in severe ARDS patients as it can yield high tidal volumes and dorsal overdistention independently from the presence of breath-staking [82], and may injure the diaphragm in case of intense effort [72].

## Additional explanations for diaphragm dysfunction in critically ill patients

### Myopathic effects of systemic inflammation and sepsis

The role of infection as a contributor to diaphragm dysfunction and the molecular mechanisms involved in such interaction have been evaluated in several studies [1, 83, 84]. Multiple other mechanisms contributing to contractile weakness besides atrophy have been identified (Fig. 1).

#### Evidence from animal models

Sepsis drives a primary inflammatory myopathy through coordinated molecular injury pathways rather than disuse alone [85]. A strong body of evidence from several animal models demonstrates that diaphragm contractile performance is profoundly reduced by sepsis and infection [86–92]. Early muscle dysfunction includes reduction in muscle-specific force generation occurring before fiber atrophy: this is mediated by cytokine-driven oxidative and nitrosative stress with direct modification of contractile proteins, which ultimately impairs force generation [93–95]. In parallel, sepsis activates upstream proteolytic systems, which disrupt sarcomeric integrity and initiate proteasome-dependent degradation [96]. Mitochondrial dysfunction further contributes through impaired oxidative phosphorylation, depletion of electron transport chain components, and reduced ATP availability, creating a bioenergetic constraint [97]. In an experimental study in rats, Ebihara and colleagues demonstrated that controlled mechanical ventilation during sepsis largely prevented sarcolemmal injury and improved diaphragm force as compared to spontaneous breathing, despite persistent elevations in oxidative stress markers and inducible nitric oxide synthase (iNOS) expression [98]. Mechanical ventilation thus mitigated the synergistic injury caused by combined oxidative and mechanical stresses during sepsis/inflammation. These findings suggest that the benefit of mechanical ventilation during high inflammatory states may primarily be due to decreased biomechanical load on a fragile diaphragm which is vulnerable to oxidative damage [99], rather than attenuation of the oxidative stress itself. Overall, this evidence supports the role of controlled mechanical ventilation in *protecting* the diaphragm during the acute phase of critical illness, a condition in which the muscle may benefit from a short period of rest. However, more prolonged sessions of controlled ventilation in a similar setting yielded opposite results [100].

#### Evidence from ventilated patients

**Fig 3.**
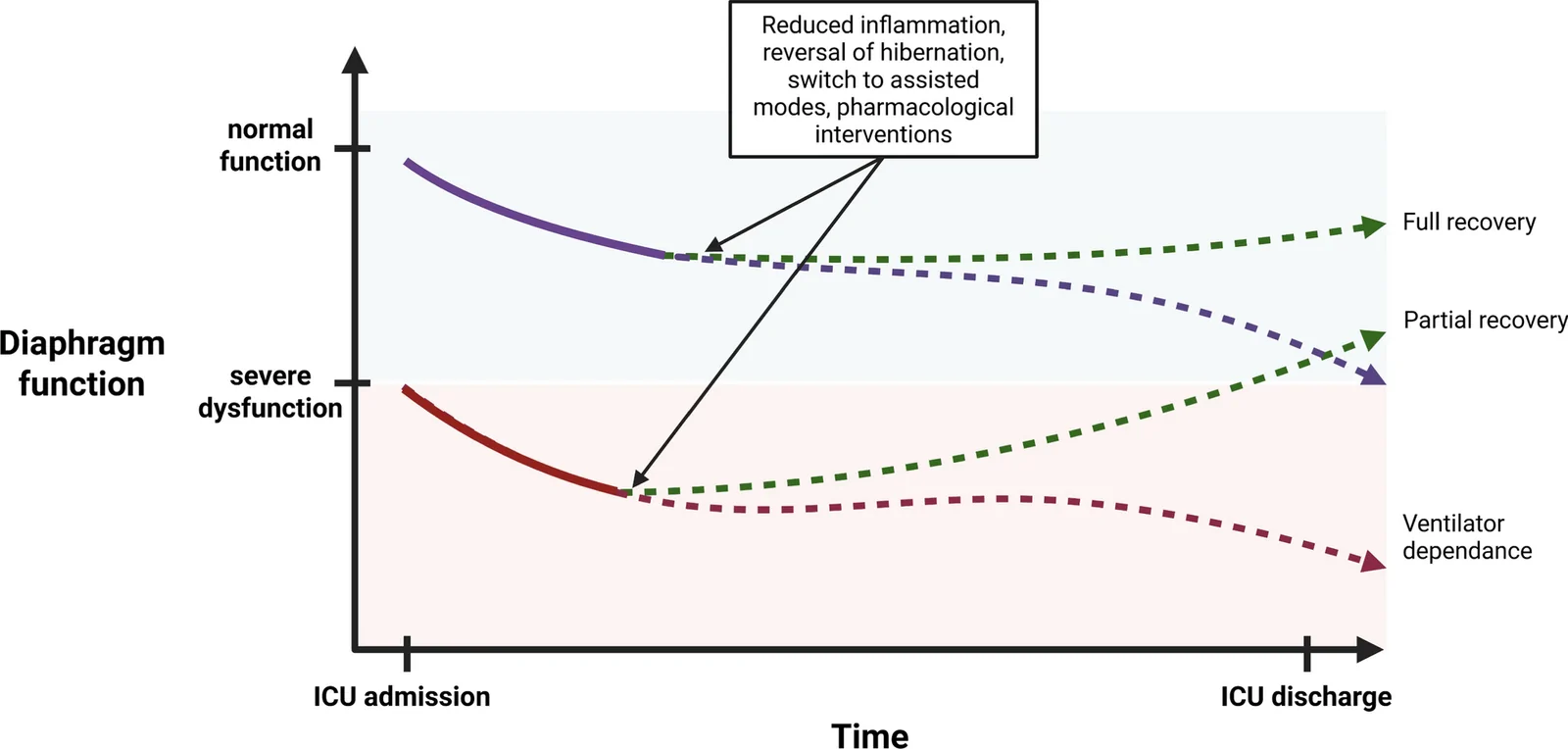
Trajectories in diaphragm (dys)function over time among ICU patients. The figure highlights the variability in diaphragm (dys)function trends among ICU patients. Patients can present with either normal or impaired diaphragm function at ICU admission, and later follow diverging trajectories (dashed lines). Several factors including baseline disease resolution, inflammatory profile, ventilatory management, pharmacological therapies and muscle training may variably affect diaphragm function recovery, which in turn has an impact on clinical outcome. Importantly, turning points can occur at variable times over the clinical course

In a seminal study, Demoule et al. demonstrated that diaphragm dysfunction was remarkably common at ICU admission, affecting approximately 60% of patients [2]. This occurred before significant exposure to mechanical ventilation, indicating that factors intrinsic to critical illness, such as sepsis, inflammation, shock and multiorgan failure, play a major part in the early development of diaphragm weakness. Similar findings were shown by Jaber et al. [31]. More recently, Lecronier et al. demonstrated in a secondary analysis of two prospective observational studies that septic patients show more severe, but reversible, impairment in diaphragm function as compared to non-septic controls, in spite of mechanical ventilation [6]. In this study, diaphragm function was measured as the pressure generating capacity of the muscle in response to magnetic phrenic nerve stimulation. Two assessments were performed: the first within 24 hours from intubation, the second within 24 hours from the switch to pressure support mode. Septic patients, with lower baseline pressure generating capacity, showed a 19% improvement after a few days, whereas that of non-septic controls was reduced by 7%. Interestingly, diaphragm thickness and SOFA scores decreased in both groups. These findings underscore the importance of sepsis and inflammation in diaphragm function modulation and the role of critical illness in its complexity as the primary driver of the problem. Indeed, diaphragm dysfunction reversibility seems to be dependent on the resolution of its initial cause, a concept perfectly in line with any kind of organ failure recovery (Fig. 3).

### Muscle hibernation

The association between sepsis/inflammation (and its resolution) with changes in diaphragm function [2, 6] and the identification of patients subgroups with opposing trajectories over time suggest the existence of reversible etiologies of diaphragm dysfunction in ventilated patients [6] (Fig. 3). Recently, it has been shown that inactivity and metabolic stress may promote the so-called super-relaxed state of myosin in the diaphragm (i.e. hibernation, a state in which inactive myosins use significantly lower amounts of ATP), a condition with unclear relationship with duration of mechanical ventilation [38]. This process, mechanistically similar to myocardial hibernation and stunning, might help preserve energy and limit proteolysis. This raises the possibility that controlled diaphragmatic rest, which could promote a myosin’s super-relaxed state [38], may be beneficial [101]. Van den Berg et al. revealed the increased percentage of super-relaxed myosins in diaphragm fibers of ventilated critically ill patients as compared to controls undergoing elective surgery [38]. This phenomenon was associated with hypo-phosphorylation of myosin regulatory light chains, and seemed to affect the diaphragm more than other muscles. The potentially protective metabolic role of diaphragm hibernation may help explain the lack of mitochondrial dysfunction and oxidative stress observed in critically ill patients (differently from brain-dead organ donors) [37]. Given the role of ischemia and tissue hypoxia in the pathophysiology of myocardial hibernation, it could be speculated that similar mechanisms, caused especially by shock states, hypoxemia, inflammation and sepsis, but possibly also by mechanical ventilation [102], may modulate the development of diaphragm hibernation as well.

Studies conducted on hibernating animals highlighted their remarkable resistance to muscle disuse atrophy despite prolonged immobility and starvation [103], with the diaphragm even exhibiting hypertrophy in some cases [104, 105]. Similarly to what described for the diaphragm of ventilated patients, skeletal muscles fibers of hibernating animals were characterized by decreased phosphorylation of myosin light chain and a reduction in ATP turnover [106, 107]. Transcriptional, metabolic, and structural adaptations aimed at reducing myosin ATP consumption including the switch to a super-relaxed state of myosin may, potentially, constitute physiological responses to particular stresses to preserve muscle mass and integrity.

Direct evidence of diaphragm hibernation *in vivo* is not available yet: nevertheless, the improvement in diaphragm function over time observed in ventilated patients with sepsis [6] could be consistent with underlying muscle hibernation (and its reversal).

### Reduced calcium sensitivity of force

Impaired calcium sensitivity of force is a well-established contributor to contractile dysfunction during muscle loading [108, 109]. This phenomenon is clinically relevant, as it was shown to affect diaphragm myofibers from patients with COPD [110] and from patients with critical illness [36], helping explain their respiratory muscle weakness and impaired contractility (Fig. 1).

In cardiac myofibers, the calcium sensitizer levosimendan acts by stabilizing the calcium-bound conformation of the troponin complex, thereby enhancing the interaction between calcium and troponin C during muscle activation and improving contractile function without increasing intracellular calcium levels [109]. Interestingly, levosimendan has been shown to increase the calcium sensitivity of force of diaphragm fibers from both COPD and non-COPD patients [111], as well as from animal models of heart failure [112], resulting in enhanced force-generating capacity. In healthy subjects, levosimendan was useful to improve diaphragm neuromechanical efficiency and force of contraction, providing indirect evidence for the occurrence of calcium desensitization during muscle loading even in the absence of overt disease [113]. However, the same effect was not observed in patients undergoing weaning from mechanical ventilation, although levosimendan did increase minute ventilation and tidal volume and reduced arterial partial pressure of carbon dioxide [114]. These findings collectively support the concept that impaired calcium sensitivity of force is a key mechanism of contractile dysfunction in the diaphragm, and that pharmacological interventions to improve calcium sensitization may be a strategy to partially restore contractile performance under certain conditions. A randomized controlled trial evaluating the use of levosimendan to facilitate weaning in critically ill patients is currently underway (ClinicalTrials.gov ID NCT07105202).

### Metabolic factors

Numerous metabolic factors are also involved in the development of diaphragm dysfunction, and should not be overlooked. Electrolyte imbalances—including hypophosphatemia, hypomagnesemia, hypokalemia, and hypocalcemia—can impair diaphragmatic contractility, as these ions are essential for neuromuscular transmission and muscle fiber physiology [115, 116]. Hypothyroidism is associated with reversible reduction in diaphragmatic strength, contributing to fatigue and respiratory failure [117]. Malnutrition, particularly protein-calorie deficiency, leads to muscle wasting, which directly reduces diaphragmatic strength and endurance [115]. Hyperglycemia, especially in the context of critical illness or diabetes, exacerbates oxidative stress and impairs mitochondrial function in diaphragm muscle fibers, resulting in impaired contractility and increased susceptibility to fatigue [118]. Finally, prolonged use of corticosteroids is linked to steroid-induced myopathy, which preferentially affects proximal muscles, including the diaphragm, and can cause significant weakness especially if combined with disuse [119]. Correction of these metabolic and drug-induced disturbances is essential for restoring diaphragmatic strength and improving respiratory outcomes, and their presence should be always considered when muscle dysfunction is encountered in the ICU.

## Is mechanical ventilation an explanation for clinically relevant diaphragm dysfunction?

**Fig 4.**
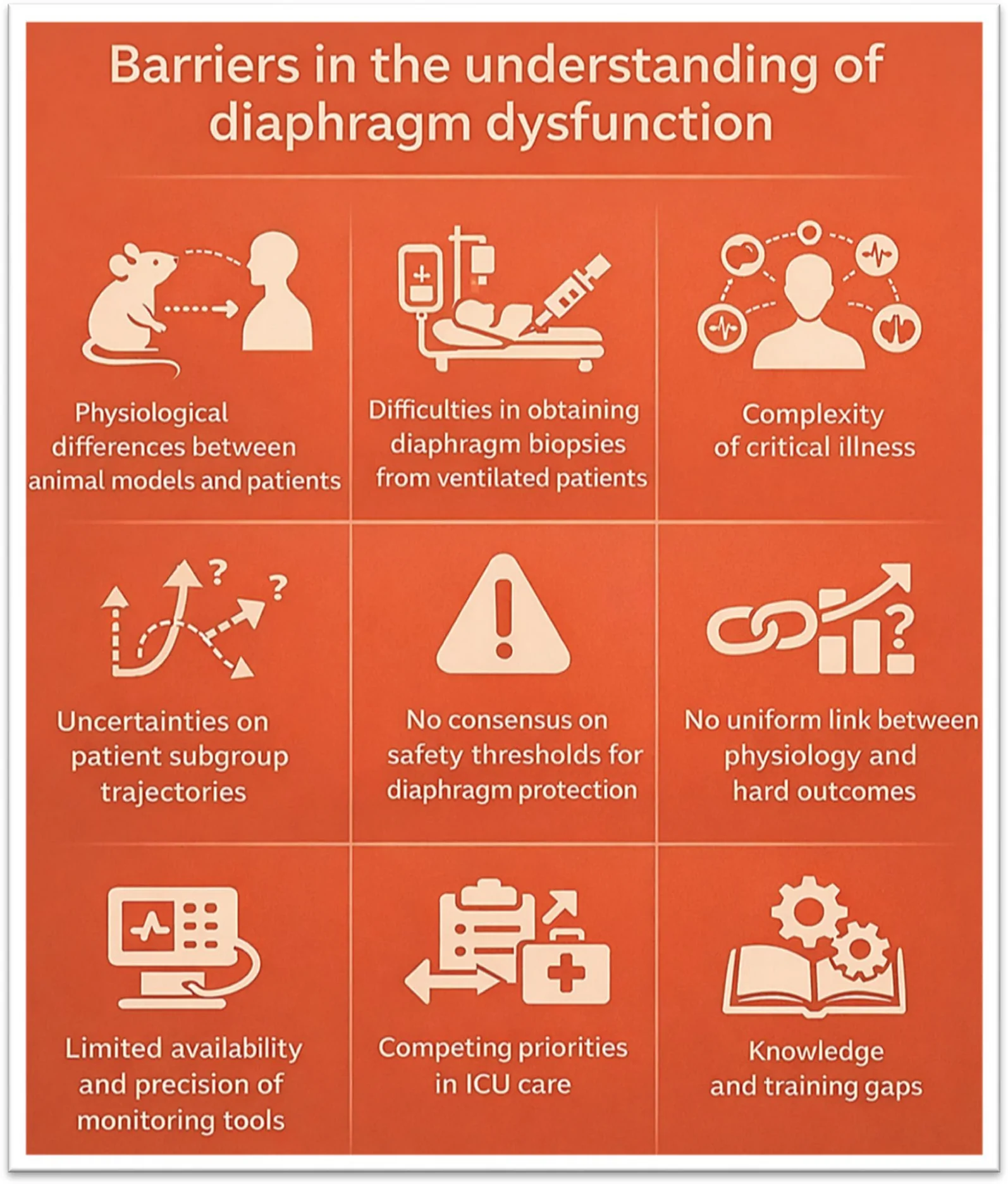
Barriers in the understanding of diaphragm dysfunction in critically ill patients. The panel highlights several key-issues in the understanding of diaphragm physiology during critical illness, affecting translation of findings from animal models to patients, and from physiological to clinical perspectives

While mechanical ventilation certainly has an impact on diaphragm structure and function, its clinical relevance, if any, is difficult to isolate and remains to be established. As outlined above, the pathophysiology of diaphragm weakness is very complex and multiple factors seems to play a role (Fig. 4). It is clear that in a subset of patients diaphragm function is already compromised at the onset of mechanical ventilation [2, 31] and that it may improve before mechanical ventilation is discontinued [6]. Therefore, at least for a portion of critically ill patients, the role of mechanical ventilation itself may be limited. In the animal setting, in which the impact of mechanical ventilation can be more easily isolated, disuse atrophy seems to play an important role in the development of diaphragm weakness, although questions remain concerning the potential for reversibility of the phenomenon and its timing. In ventilated patients (excluding brain-dead organ donors), given the greater overall complexity, no studies were able to successfully achieve the same goal.

If the role of the ventilator in the pathophysiology of diaphragm weakness in critically ill patients is limited or at least unclear, clinicians should be careful in adopting a strategy of diaphragm protective ventilation, especially when this requires higher level of sedation or the reinstitution of fully controlled ventilatory modalities. First, the risks of maintaining fully controlled mechanical ventilation vs. transitioning to assisted breathing should be comprehensively assessed on different levels including lung protection, preservation of cognitive function and prevention of dyspnea. Second, the monitoring and the modulation of inspiratory effort during assisted breathing according to cutoff values mostly used in the context of lung protection should be carefully evaluated. Indeed, recent observational studies further questioned the association between inspiratory effort, diaphragm injury and clinical outcomes. Grassi and colleagues showed that intensity of patient effort was not independently associated with mortality, although median effort levels were relatively low, reflecting the expertise of participating centers in managing assisted ventilation [120, 121]. A recent work by Dianti et al. suggested that the relationship between inspiratory drive and effort with mortality is strongly determined by lung function, with associations between high effort levels and poor prognosis confined to patients with moderate-to-severe hypoxemia (PaO_2_/FiO_2_ < 150 mmHg) [122]. These results may imply that priority should be given to lung-, rather than diaphragm-protection when titrating mechanical support, considering “ideal” effort levels primarily in the context of lung protection [123]. Indeed, both load-induced and under-assistance diaphragm myotrauma remain largely theoretical constructs in the ICU: their existence, detection, modifiability and impact have yet to be convincingly demonstrated. Moreover, the achievement of ‘diaphragm-protective’ ventilation would often require interventions that may introduce additional risks, such as deeper sedation, partial neuromuscular blockade, or extracorporeal support strategies for CO_2_ removal or oxygenation [18, 124–127]. We therefore believe that the current evidence on load-induced diaphragm myotrauma is still too limited to guide diaphragm-centered clinical decisions in critically ill patients at the expense of something else. On the other hand, we deem reasonable to advocate the adoption of personalized ventilatory settings to minimize the risk of lung injury, including the modulation of inspiratory effort, and avoid under- and over-assistance of the respiratory muscles [14, 19].

Taken together, these considerations warrant caution in assuming that mechanical ventilation is the main contributor to diaphragm dysfunction, with controlled rest potentially playing a diaphragm protective role during the most acute phase of critical illness (e.g. when diaphragm hibernation is present) [98, 101]. We therefore believe that the term *critical illness-* (rather than *ventilator-*) *associated* diaphragm dysfunction better defines and characterizes the condition (Fig. 1).

## Clinical implications

In clinical practice in the intensive care unit, attention should be paid to identify and treat any potentially reversible cause of diaphragm dysfunction, considering the complexity of its pathogenesis. While keeping in mind that ventilator support per se may not be the main culprit of the problem, inspiratory effort levels considered ‘diaphragm protective’ were shown to be associated with improved outcome in more hypoxemic patients [122]. However, whether the commonly considered ‘safe’ levels of inspiratory effort between 3 and 12 cmH_2_O of Pmus are ideal for all patients remains to be determined, especially from a diaphragm perspective (as not only the diaphragm, but all respiratory muscles may contribute to the generation of Pmus) and in conjunction with other interventions such as PEEP and pressure support modulation, sedation and pharmacological or extracorporeal therapies [16, 18]. Indeed, the observed benefit associated with these strategies may reflect the combined effects of reduced stress and strain to the lungs, lower use of sedatives and neuromuscular blockers and all interventions associated with earlier mobilization and faster recovery trajectories.

An integrated monitoring of respiratory mechanics during assisted breathing with indexes of inspiratory effort and lung distending pressures remains paramount [128], but a careful evaluation of pros and cons of any further intervention aimed at targeting ‘safe’ thresholds is warranted. When the pursue of the so-called *diaphragm protective* range is conflicting with other patient-centered interventions (e.g. need for higher sedation) and/or is not favoring lung protection as well, it could probably be abandoned.

## Future directions

As a means to avoid and potentially revert muscular disuse atrophy, diaphragm pacing through transvenous, transcutaneous or magnetic phrenic nerve stimulation sessions is under active investigation, with the goal of facilitating weaning (Fig 1). However, evidence so far is limited and the topic controversial, also because of safety concerns [129–132]. Moreover, questions remain regarding the timing for the intervention over the course of disease and the precise ventilatory targets during its application [101]. If some degree of diaphragm rest were useful in the most acute phase of disease to protect the muscle, diaphragm pacing could possibly even become deleterious: proving the occurrence of diaphragm hibernation in critically ill patients would help clarify this complicated issue [101].This could be achieved by longitudinally monitoring diaphragm function throughout disease course, aiming at identifying distinct patient sub-phenotypes whose diaphragm function trajectories behave (or can be modulated) differently, in more personalized ways.

Finally, pharmacological interventions directly aimed at restoring muscle efficiency seem interesting and promising: these would include the use of calcium sensitizers [114, 133], troponin activators [38] and, possibly, immune-modulators [134].

## Conclusions

In conclusion, we strongly support the theory in which diaphragm dysfunction in mechanically ventilated patients is primarily a manifestation of critical illness rather than a direct consequence of mechanical ventilation. While excessive or insufficient ventilator assistance has been shown to result in diaphragm disuse atrophy or overload in animal models, data in humans are less clear [35, 37, 38, 42]. Diaphragm dysfunction often precedes the initiation of mechanical ventilation [2, 31] and its recovery parallels the resolution of the underlying condition, implying the existence of reversible mechanisms [6, 36, 38, 84]. Therefore, the long-standing concept of *ventilator-induced diaphragm dysfunction*, commonly endorsed even in clinical settings [13], may oversimplify a far more complex and multifactorial condition. Recognizing *critical illness–associated diaphragm dysfunction* as a more comprehensive and accurate framework could help refocus research and implement more effective clinical strategies.

## Data Availability

Not applicable.

## Abbreviations

ARDS: Acute respiratory distress syndrome
COPD: Chronic obstructive pulmonary disease
ICU: Intensive care unit
iNOS: Inducible nitric oxide synthase
MRI: Magnetic resonance imaging
PEEP: Positive end-expiratory pressure
Pmus: Respiratory muscle pressure
VIDD: Ventilator-induced diaphragm dysfunction
